# Depression trajectories during the COVID-19 pandemic: a secondary analysis of the impact of cognitive-appraisal processes

**DOI:** 10.1186/s41687-023-00600-z

**Published:** 2023-07-13

**Authors:** Carolyn E. Schwartz, Katrina Borowiec, Bruce D. Rapkin

**Affiliations:** 1 DeltaQuest Foundation, Inc., 31 Mitchell Road, Concord, MA 01742 USA; 2 Departments of Medicine and Orthopaedic Surgery, Tufts University Medical School, Boston, MA USA; 3 Department of Measurement, Evaluation, Statistics, and Assessment, Boston College Lynch School of Education and Human Development, Chestnut Hill, MA USA; 4 Department of Epidemiology and Population Health, Albert Einstein College of Medicine, Bronx, NY USA

**Keywords:** Appraisal, Depression, Chronic illness, General population, Response shift, COVID

## Abstract

**Purpose:**

This study characterized depression trajectories during the COVID pandemic and investigated how appraisal and changes in appraisal over time related to these depression trajectories.

**Methods:**

This longitudinal study of the psychosocial impact of the COVID-19 pandemic included 771 people with data at three timepoints over 15.5 months. The depression index was validated using item-response-theory methods and receiver-operating-characteristic curve analysis. The Quality of Life (QOL) Appraisal Profile_v2_ Short-Form assessed cognitive-appraisal processes. Sequence analysis characterized depression-trajectory groups, and random effects models examined appraisal main effects, appraisal-by-group, and appraisal-by-group-by-time interactions.

**Results:**

Sequence analysis generated six trajectory groups: Stably Well (n = 241), Stably Depressed (n = 299), Worsening (n = 79), Improving (n = 83), Fluctuating Pattern 1 (No–Yes–No; n = 41), and Fluctuating Pattern 2 (Yes–No–Yes; n = 28). While all groups engaged in negative appraisal processes when they were depressed, the Stably Depressed group consistently focused on negative aspects of their life. Response-shift effects were revealed such that there were differences in the appraisal-depression relationship over time for standards of comparison and recent changes for the Stably Depressed, and in health goals for those Getting Better.

**Conclusion:**

The present work is, to our knowledge, the first study of response-shift effects in depression. During these first 15.5 pandemic months, group differences highlighted the connection between negative appraisals and depression, and response-shift effects in these relationships over time. Egregious life circumstances may play a lesser role for the Stably Depressed but a greater role for people who have transient periods of depression as well as for those with improving trajectories (i.e., endogenous vs. reactive depression). How one thinks about QOL is intrinsically linked to mental health, with clear clinical implications.

**Supplementary Information:**

The online version contains supplementary material available at 10.1186/s41687-023-00600-z.

## Introduction

Depression is one of the most common mental disorders in the United States (US), affecting about eight percent of American adults at a given time [1]. Twice as common among women as among men [1], its prevalence is associated with financial strain [1] and substance use [2, 3]. Depression can result in severe impairment in one’s ability to carry out major life activities [4], and it increases the risk of disability in late life [5].

Depression may play an even greater role in exacerbating the impact of chronic medical conditions. Many chronic medical illnesses include depression symptoms and may be comorbid with major depression. Such combined effects of physical and mental symptoms may lead to higher levels of disability [6], and that sadness exacerbates physical symptoms such as fatigue [7]. Depressive symptoms have been implicated in a reduction in health-enhancing goals, and thus may impact the adoption and maintenance of healthy lifestyles [8].

Successful non-pharmacologic interventions for depression include cognitive-behavioral therapy, by which individuals are taught to identify and modify disempowering self-talk [9]. Such self-talk would emphasize the durability, uncontrollability, and self-blame for negative life events [10]. Cognitive Behavioral Therapy involves efforts to change thinking patterns by recognizing distortions in thinking that create problems, gaining a better understanding of others’ behavior and motivation, using problem-solving skills, and developing greater confidence in one’s abilities [11]. The therapeutic focus is on facing one’s fears, using role-playing to prepare for difficult interactions with others, and learning to calm oneself and relax one’s body [11].

There may, however, be other types of self-talk that are relevant to mitigating depression’s impact. For example, emerging research on cognitive-appraisal processes has revealed that the way patients think about health and quality of life (QOL) can influence how burdensome they perceive their treatment [12] and thus their adherence to treatment [13]. It can influence how they recognize change in pain or disability [14] and their perception of quality of medical care [15]. Understanding how individuals with depression symptoms differ in their thinking about health and QOL (i.e., cognitive-appraisal processes) may be relevant to identifying additional promising pathways to intervention (i.e., via cognitive-behavioral therapy aimed at modifying such appraisals). In other words, the cognitive-appraisal processes related to health and QOL may be distinct from the self-talk that has been linked to depression (i.e., durability, uncontrollability, and self-blame [10]), but may be pertinent to repercussions from these same tendencies. Expanding the realm of cognitions that are the focus of such interventions may be useful for helping individuals coping with medical challenges.

In addition to intervening to reduce depression, such information about cognitive-appraisal processes is relevant to person-centered medical care. Dovetailing with the USA Food and Drug Administration’s recent emphasis on measuring patient experience [16, 17], the past seven years have witnessed what some have called an “unprecedented collaboration of change agents working across organizations and communities transforming the way we think and act to advance health, well-being, and equity globally” [18]. Healthcare systems in over 40 countries have aligned with a person-centered approach that emphasizes a compassionate focus on the person [18]. Moving away from asking “What’s the matter?” to asking, “What matters to you?” [19], this focus leads to a consideration of a broad range of factors that can influence the success of medical care. This consideration brings to the foreground how lack of sociodemographic resources can degrade patient engagement and adherence [20]. It also highlights how cognitive-appraisal processes inform key constructs in quality-of-care assessment, such as satisfaction with care, experiences of care, and problem resolution, and how these key constructs are driven by an individual’s context and resources [15].

The present study follows up on a decade-long evolution by addressing a research question formulated by patients and researchers at multiple conference presentations. The question was “Are depressed people less likely to engage in response shifts?” Response shift is defined as changes in an individual’s evaluation of QOL after a health-state, life-circumstance, or developmental change (catalyst) that relates to changes in values (recalibration), priorities (reprioritization), or conceptualization (reconceptualization) of QOL [21–24]. We operationalize response shift using measures of cognitive appraisal that assess four appraisal parameters deemed relevant to patient self-report on QOL: frame of reference, sampling of experience, standards of comparison, and combinatory algorithm (i.e., patterns of emphasis) [23–25]. In the Rapkin and Schwartz QOL Appraisal model, the “three R’s of response-shift” are operationalized as follows: Reconceptualization is change in frame of reference; Recalibration is change in standards of comparison; and Reprioritization is change in sampling of experience and/or combinatory algorithm. In statistical modeling to test response-shift hypotheses, response shift is inferred when changes in appraisal explain QOL change over time after a catalyst, after adjusting for clinical and demographic covariates [23–25]. Response shift is generally considered a positive adaptation, one that supports resilience [26, 27], although Li and Rapkin found evidence of both positive and negative response-shift effects on mental health [28]. The present study thus sought to characterize depression trajectories over about 15.5 months of follow-up during the COVID pandemic (catalyst), and to investigate how appraisal and changes in appraisal over time related to these depression trajectories.

## Methods

### Sample and design

This secondary analysis utilized data collected for a longitudinal study of the psychosocial impact of the COVID-19 pandemic. Following Schwartz et al.’s published guidelines for secondary analysis for response-shift detection [29], we describe the study whose data was utilized for this secondary analysis. The original study aimed to identify patient factors that confer resilience to the COVID pandemic, focusing on COVID-specific cognitive, behavioral, and demographic aspects, as well as more broadly relevant factors such as QOL appraisal and reserve-building activities. The primary difference in this secondary analysis is that it focused specifically on depressive symptoms as an outcome, rather than the broader multidimensional QOL outcomes included in the full dataset.

The study recruited participants via Rare Patient Voice and Ipsos Insight —the former to target patients and caregivers of people with chronic medical conditions; the latter to target a general-population sample of US adults who were heterogeneous in terms of health. This general-population subsample was recruited to yield an overall sample that was more diverse and more nationally representative in terms of age distribution, gender, region, and income. The study sample included 771 individuals, of whom 527 were patients, 91 were caregivers, 29 were patient-caregivers, and 124 were neither. Data were collected at three time points: baseline (late Spring 2020), follow-up 1 (Spring 2021), and follow-up 2 (Fall 2021). Table 1 provides the sociodemographic characteristics of the study sample.

**Table 1:** Demographic characteristics of study participants at baseline (n = 771)

Variable	%
Gender: % female	82
Marital status	
Never married	15
Married/cohabiting	63
Separated/divorced	16
Widowed	6
Hispanic ethnicity	1
White race	94
Had COVID	5
Education	
High school diploma or less	7
Trade or technical school	6
Some college	23
Bachelors degree	34
Graduate or professional degree	29
Employment Status at Baseline	
Currently working	36
Unemployed	11
Retired	30
Disabled due to medical condition	23
Difficulty paying bills	
Not at all	59
Slightly	22
Moderately	10
Very	6
Extremely	3

	Mean	SD
Age	55.56	13.45
No. of comorbid conditions*	3.31	2.13
Years since diagnosis	15.28	13.64
Body Mass Index	29.57	8.53

^*^Excluding depression

Participants were not paid for their participation, although Ipsos Insight used its usual respondent point-related incentives. Eligible participants were age 18 or older and able to complete an online questionnaire. Participants with motor, visual, and/or other problems that made it difficult for them to complete the web-based survey enlisted the assistance of someone else to enter the participant’s answers. This survey was administered through the secure Alchemer engine (www.alchemer.com), which is compliant with the US Health Insurance Portability and Accountability Act. The protocol was reviewed and approved by the New England Independent Review Board (NEIRB #2021164), and all participants provided informed consent prior to beginning the survey.

### Measures

*Depression* was measured by a depression index created using items from existing measures that reflected similar content to the Patient Health Questionnaire-8 (PHQ-8) [30], a validated and commonly-used depression measure, as well as content that depressed patients endorsed as very important to determining remission from depression [31]. This approach is similar to one taken by Kubzansky and colleagues who sought to examine emotional vitality in a secondary analysis of existing data [32]. Our 14-item depression index included three items from the Patient-Reported Outcome Measurement Information System-10 (PROMIS-10) [33], one from the NeuroQOL Applied Cognition [34], eight from the NeuroQOL Positive Affect and Well-Being [34], and two from the Ryff Environmental Mastery subscale [35]. Additional file 1: Table S1 provides item content and source for the items included in the Depression Index. Additionally, patient-reported information at baseline on depression as a comorbidity were used to create a meaningful cut-point for this depression index (see Statistical Analysis below). The items included in this measure reflect depressive symptoms, and henceforth we shall refer to the Depression Index score as “depression” for ease of exposition of the study results.

*Cognitive-appraisal processes* were assessed using the QOL Appraisal Profile_v2_ Short-Form. This short form contains items assessing patterns of emphasis (i.e., Combinatory Algorithm; 9 items), Standards of Comparison (9 items), Sampling of Experience (4 items), and Goal Delineation [36]. Response options on the Likert-scaled measure range from one to five, with response options endorsing agreement (strongly disagree to strongly agree) for Combinatory Algorithm, frequency (never to always) for Standards of Comparison and Sampling of Experience, and similarity to themselves (not at all like me to very much like me) for Goal Delineation. All items also allow respondents to decline to answer (“Do not know/does not apply to me”). This measure has been used to investigate the cognitive-appraisal processes underlying QOL assessment [36–38], to investigate group differences in aspirations [39, 40], and to characterize a “personalized medicine” approach for longitudinal orthopaedic outcome research [41–43]. The QOLAP is an “idiometric” measure, meaning that psychometric characteristics of the measure are contingent on contextual or situational information [44, 45]. While the QOLAP items can be included in analyses as individual items [43, 46], this approach leads to a large number of comparisons. The present work thus utilized the data-reduction approach utilized in many past studies where principal component scores are created to maximize explained variance in the sample and reduce the number of comparisons to avoid false-positive findings [44].

*Demographic characteristics* included age, gender, years since diagnosis, race, ethnicity, education, financial hardship (operationalized as difficulty paying bills), employment status, cohabitation/marital status, height and weight (to compute body mass index), comorbidities (excluding depression), and whether the individual had been infected with Sars Cov-2.

*Time* was measured as both a categorical indicator (i.e., baseline, follow-up 1, follow-up 2) and as a continuous measure of days since baseline data collection.

### Statistical analysis

#### Overview

As noted earlier, the present study sought to characterize depression trajectories over about 15.5 months of follow-up during the COVID pandemic, and to investigate how individual differences and changes in appraisal over time related to these depression trajectories. The COVID pandemic is conceptualized as a likely catalyst of response shifts. The widespread impact of COVID provided an opportunity to better understand how depressive symptoms do or do not emerge and evolve in relationship to cognitive appraisal. The underlying idea is that people may fluctuate from being depressed to not being depressed over time, and that capturing their individual trajectories and characterizing them may help to understand what cognitive-appraisal processes are associated with these health-state changes.

**Fig. 1:**
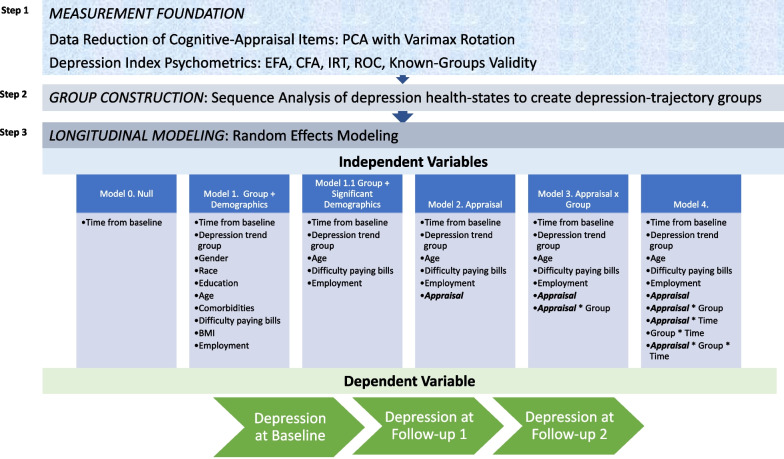
Summary of the three steps of statistical analysis implemented. The first step was a careful measurement foundation comprised of psychometric analyses to create the Depression Index, and data-reduction techniques to create robust and valid indicators or summaries of distinct appraisal processes. The second step involved group construction which comprised creating distinct depression-trajectory groups on the basis of the respondents’ path between health states (depressed vs. not depressed) over the three follow-up time points. Finally, the third step involved longitudinal modeling, which applied an iterative series of random effects models of these time-varying (e.g., appraisal) and time-invariant (e.g., group) indicators to investigate how appraisal mediated and moderated the experience of depression (outcome) over time (time) as a function of depression trajectory group (group), after adjusting for demographic covariates. Note that”Appraisal” is used to represent each of the six appraisal composite-score variables

The work thus involved multiple steps summarized in Fig. 1. The first step was a careful measurement foundation comprised of psychometric analyses to create the Depression Index, and data-reduction techniques to create robust and valid indicators or summaries of distinct appraisal processes. The second step involved group construction which comprised creating distinct depression-trajectory groups on the basis of the respondents’ path between health states (depressed vs. not depressed) over the three follow-up time points. These depression-trajectory categories allowed us to examine associations between depression and appraisal among groups defined by how their experiences during the pandemic unfolded. Finally, the third step involved longitudinal modeling, which applied an iterative series of random effects models of these time-varying (e.g., appraisal) and time-invariant (e.g., group) indicators to investigate how appraisal mediated and moderated the experience of depression (outcome) over time (time) as a function of depression trajectory group (group), after adjusting for baseline demographic covariates. The longitudinal modeling addressed main effects of appraisal, two-way interactions of appraisal-by-time, and three-way interactions of appraisal-by-group-by-time.

Thus, the depression-trajectory groups represent a higher level of abstraction, not merely depression or time. These groups allowed us to untangle associations between depression and appraisal among people who had similar levels of depressive symptoms at a given point in time, but who ultimately were on different health-state sequences or paths. Because the created depression trajectory groups inherently reflect group-by-time interactions on depression, we did not focus on interpreting these two-way interactions, but had to include them in the model in order to test for appraisal-by-group-by-time interactions. This three-way interaction essentially asks whether the growth curve of the depression-trajectory groups differed as a function of appraisal. This was the only way to get at group differences in appraisal over time (i.e., response-shift effects by group).

### Measurement foundation

#### Depression index

We began by creating a depression index using the abovementioned 14 items with similar content to the PHQ-8. Items were (re)coded such that high scores reflected worse depression. Exploratory factor analysis using principal axis factoring with no rotation was done on a randomly selected 50% of the baseline sample using SPSS, and confirmatory factor analysis on the remaining 50% of the baseline sample using Mplus to generate model-fit statistics. The data fit a bifactor model, with one general and two specific factors (details provided under Results). We then used the general factor for the depression index, creating an IRT-based score using a bifactor graded response model [47] specification with IRTPRO. Receiver Operating Characteristic Curve analysis [48] was used to classify individuals depending on levels of true- and false-positive rates, in order to select a cut-point for subsequent analyses of depressed versus non-depressed patients. Internal consistency reliability was computed for the index, and known-group validity of the Depression Index was tested with independent sample t-tests in SPSS that compared mean Depression Index scores (dependent variable) for those who endorsed depression as a self-reported comorbidity vs. not (independent variable). For the appraisal items, we sought to reduce the number of statistical comparisons using principal components analysis (PCA) with varimax rotation so that the resulting composite scores were orthogonal to each other.

#### Data-reduction of the QOLAPv2

A principal components analysis (PCA) with a Varimax rotation with Kaiser Normalization was done on the cognitive-appraisal items using SPSS. An eigenvalue of 1.0 was the cut-off for inclusion of a component in the resulting component scores. Because the appraisal domains are multidimensional, not variables, the resulting components generated are not expected to reduce to four scores reflecting each of the four aspects of cognitive appraisal. Consistent with appraisal theory and idiometric measures, PCA is used for each study sample rather than generating scores derived from earlier studies, because we do not expect to identify consistent latent factors underlying a set of items that pertain in every situation [44].

### Group construction

#### Creating depression-trajectory groups

Stata’s sequence analysis was used to identify depression-trajectory groups based on whether someone was depressed or not at each of the three time points based on the ROC cut-point. This analysis uses a bundle of Stata programs (SQ-Ados [49]) to work with binary grouping data (i.e., depressed vs. not) and all time points to produce a listing of all sequences in the data set. Illustrative trajectory plots for the resulting groups were plotted using Excel.

### Longitudinal modeling

Random effects models were used to examine predictors of depression (outcome) as a function of time, trajectory group (group) and appraisal (moderator). Time was used as a continuous measure in these RE models to capture variation in the precise time point (in days) of data collection among respondents. Additionally, the regression coefficient corresponding to the time variable can be interpreted meaningfully as "for each additional day, participants' depression score was predicted to increase by the coefficient corresponding with time, holding all else constant."

The resulting trajectory groups were used in subsequent random effects (RE) modeling [50] using SPSS, examining the depression index as a continuous score (dependent variable). Independent variables were baseline demographic covariates, depression-trajectory group using the Stably Well group as the referent, and time in days (RE Model 1); appraisal composite (added in RE Model 2); appraisal*group interaction (added in RE Model 3); group*time, appraisal*time, and appraisal*group*time interactions (added in RE Model 4). Each appraisal composite was examined in its own series of random effects modeling. Because the created depression trajectory groups inherently reflect group-by-time interactions on depression, we will not focus on interpreting these two-way interactions, but must include them in the model in order to test for appraisal-by-group-by-time interactions.

In interpreting interaction effects, we relied on the Fisherian approach [51] to interpreting interactions, starting with the highest-order significant interaction first, and then going down to simpler terms. If the two-way appraisal-by-group interaction only emerged as significant in the model also including the three-way appraisal-by-group-by-time interaction but also the two-way group-by-time interaction, we would test for a possible suppression effect by testing an additional two-way model that includes group-by-time interactions. This suppression effect would, for example, suggest that the group-by-time interaction removes variance from the dependent variable that is not shared with the appraisal-by-group interaction effect, so the latter only emerges as significant when the former is controlled.

To facilitate interpretation, time was represented as baseline, follow-up 1, and follow-up 2—i.e., corresponding to an average of 0, 168, and 466 days, respectively, since baseline—in analyses generating the marginal means used to plot two- and three-way interactions. It was used as a continuous measure in RE models to account for individual variability in days since baseline for the first and second follow-ups.

Statistical analyses were implemented using IBM SPSS version 28 [52], Mplus Version 8.8 [53], IRTPRO version 6.0.4.12 [54], Stata version 17 [55], and Microsoft Excel.

## Results

### Depression index

The exploratory factor analysis on 50% of the baseline sample yielded a two-factor solution with eigenvalues greater than 1 (7.87 and 1.01, respectively). All of the items loaded on the first factor, and the factor loadings on the second factor were relatively weak (< 0.43). The first factor also explained a much higher proportion of the variance (56% vs. 7%). A forced extraction of 1 factor yielded high loadings (0.55–0.86) and a high coefficient alpha (0.94).

We then implemented a confirmatory factor analysis on the remaining 50% of the baseline sample. A one-factor model had poor fit statistics (RMSEA = 0.174, CFI = 0.946, TLI = 0.936) (CFA Model 1). Items assessing purpose and meaning were highly correlated (r = 0.98) but re-running the model dropping one of the items (purpose) did not improve model fit (RMSEA = 0.176, CFI = 0.946, TLI = 0.935) (CFA Model 2). We examined the Variance Inflation Factor to investigate collinearity. None of the items were considered problematic. We thus examined a bifactor model (CFA Model 3), which included a general depression factor score and two specific factors: (1) where all of the positively worded items loaded on one specific factor (positive affect); and (2) all the negatively worded items loaded on another specific factor (negative affect). The fit improved with this model (RMSEA = 0.093, CFI = 0.987, TLI = 0.982). In CFA Model 3, the loading of the PROMIS general mental health item on the specific factor was not statistically significant. We thus re-ran CFA Model 4 but removed this item from the positive affect specific factor. The change in fit was negligible (RMSEA = 0.092, CFI = 0.987, TLI = 0.982). We thus proceeded with using CFA Model 3 for the IRTPRO analyses.

The IRTPRO analysis was conducted on the baseline data with the bifactor model selected from the CFA. The model met the convergence criteria. The RMSEA computed by IRTPRO was 0.07, which was lower than CFA Model 3’s value reported above using 50% of the sample and implemented with Mplus. All the items had good fit (i.e., *p* > 0.01). Similar to above, the PROMIS general mental health item had a weak loading on the specific factor, which could be explained by its “general health” content. The depression index was the general depression factor score (Depression Index), and was based on the IRTPRO specifications shown in Additional file 1: Tables S1 and S2.

The ROC Curve analysis distinguished between those who had or had not endorsed having depression as a co-morbidity at baseline. The Area Under the Curve value was 0.78 (*p* < 0.0001; 95% CI: 0.75–0.81), which is considered “fair” based on interpretation guidelines provided by El Khouli et al. [56]. We selected a cut-point for our Depression Index based on Power et al.’s recommendations that the sum of sensitivity and specificity should be at least 1.5 [57]. Our highest value was 1.406 (sensitivity = 0.71, specificity = 0.70), which was represented by a score of 50 on our Depression Index (Additional file 2: Figure S1; Additional file 1: Table S3).

Known groups validity comparing the continuous Depression Index scores for those endorsing depression as a self-reported comorbidity versus not revealed significant differences in mean Depression Index scores (t = −15.26, *p* < 0.0001) a large effect size (Cohen’s d = −1.10) [58]. This finding supports the notion that this newly developed Depression Index is a valid indicator of depression.

### Data reduction of appraisal items

**Fig. 2:**
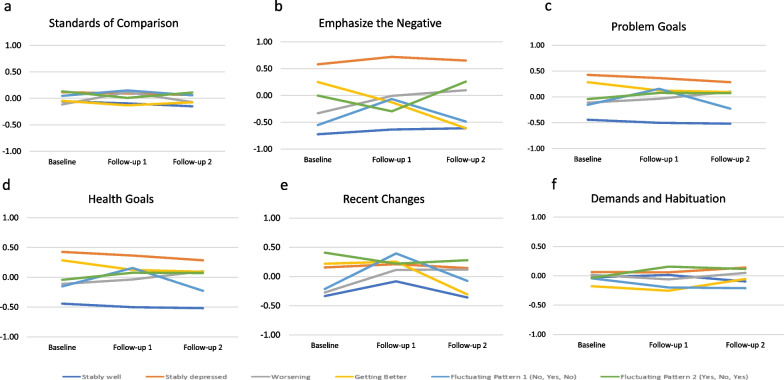
**a**–**f** Trajectory Group Patterns of Appraisal over Time. These plots display unadjusted means by depression-trajectory group on each of the six appraisal composite scores

The PCA of the appraisal items yielded six components that explained 54.94% of the variance (see Additional file 1: Table S4). The first component comprised thinking a great deal about comparisons to other people or circumstances in order to evaluate their QOL (“Focused on Comparisons”); the second focused on negative patterns of emphasis (e.g., negative, out of control, resigned)(“Emphasizing the Negative”); the third on problem goals (e.g., get out of a rut, feel settled, solve problem, get more help with multiple domains)(“Problem Goals”); the fourth on health goals (e.g., recent health problems and flare ups, get more support from providers, depend less on others)(“Health Goals”); the fifth on sampling recent events and changes (“Recent Changes”); and the sixth on sampling obligations and habituating to the way things are (Demands & Habituation). Figure 2a–f display unadjusted means by depression-trajectory group on each of the six appraisal composite scores.

### Depression trajectory groups

The sequence analysis generated eight trajectory groups (Table 2). We sought to combine groups with similar patterns, such as generally improving or generally worsening. Accordingly, we worked with the following six groups: Stably Well (n = 241), Stably Depressed (n = 299), Worsening (n = 79), Improving (n = 83), Fluctuating Pattern 1 (No, Yes, No; n = 41), and Fluctuating Pattern 2 (Yes, No, Yes; n = 28). We kept the two fluctuating pattern groups separate because their patterns were opposite one another, and they might have “cancelled each other out” in subsequent linear modeling. Additional file 2: Fig. 2a–f illustrate the individual growth trajectories for these six trajectory groups by showing a random selection of 20 participants within each trajectory group.

**Table 2:** Depression trajectory groups

Depression sequence	Time 1	Time 2	Time 3	Count	Percent	Trajectory description
111	Yes	Yes	Yes	299	39%	Stably depressed
000	No	No	No	241	31%	Stably well
110	Yes	Yes	No	43	6%	Improving
011	No	Yes	Yes	42	5%	Worsening
010	No	Yes	No	41	5%	Fluctuating
100	Yes	No	No	40	5%	Improving
001	No	No	Yes	37	5%	Worsening
101	Yes	No	Yes	28	4%	Fluctuating

0 = Not depressed using IRT cut score. 1 = Depressed

### Longitudinal modeling

The initial random effect models revealed non-significant effects of time in predicting the continuous Depression Index (Additional file 1: Table S5). Nevertheless, time remained in the model to reflect the longitudinal nature of the data and for subsequent interaction analyses. When trajectory group and demographic characteristics were added to the model, all trajectory groups remained significant, meaning that their predicted levels of depression differed from the stably well group. Additionally, younger age, higher reported difficulty paying bills, and being retired or disabled from working due to a medical condition were significantly associated with worse depression (RE Model 1). Demographic characteristics that were not significant were removed in a revised version of RE Model 1 (i.e., RE Model 1.1). Appraisal was added in RE Model 2; appraisal-by-group was added in RE Model 3; and appraisal-by-time, group-by-time, and appraisal-by-group-by-time interactions were added in RE Model 4. Separate versions of RE Models 2 through 4 were examined for each of the six appraisal composites. The referent in these analyses was the Stably Well group.

**Fig. 3:**
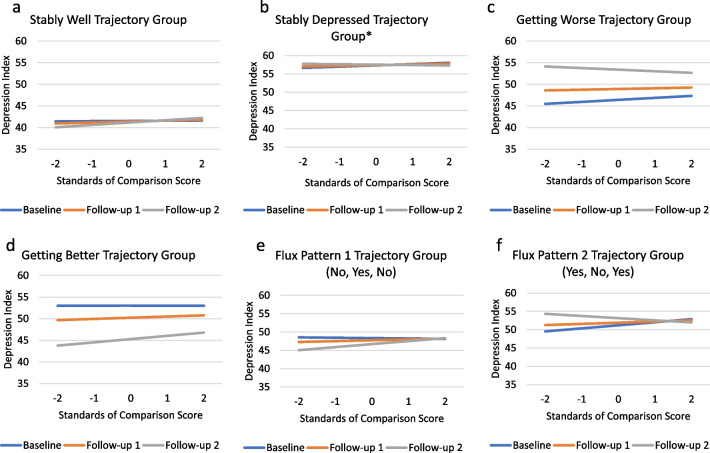
**a**–**f** Standards of Comparison Three-Way Interactions. These plots show adjusted means by depression-trajectory group illustrating the three-way interaction (appraisal-by-group-by-time) on Standards of Comparison for each data collection timepoint. Asterisks (*) indicate significant trajectory group parameter estimates

Appraisal processes characterized by focusing on comparing oneself to others (Standards of Comparison) had a non-significant main effect for appraisal, and non-significant appraisal-by-group and appraisal-by-time interactions. There was a significant three-way appraisal-by-group-by-time interaction (Fig. 3a–f) for the Stably Depressed group, such that while the Stably Depressed generally engaged in more comparison with others, the relationship to depression changed slightly from baseline to follow-up relative to the Stably Well group. For members of the Stably Well group, there was essentially no relationship between engaging in comparison with others and depression at baseline. However, at Follow-up 2, depression increased slightly as comparison with others increased. In comparison, at baseline, higher scores on Standards of Comparison were associated with higher scores on the Depression Index for members of the Stably Depressed Group, whereas by Follow-up 2, lower scores on Standards of Comparison were associated with higher Depression Index scores (Fig. 3a, b; Additional file 1: Table S5, Model 4a).

**Fig. 4:**
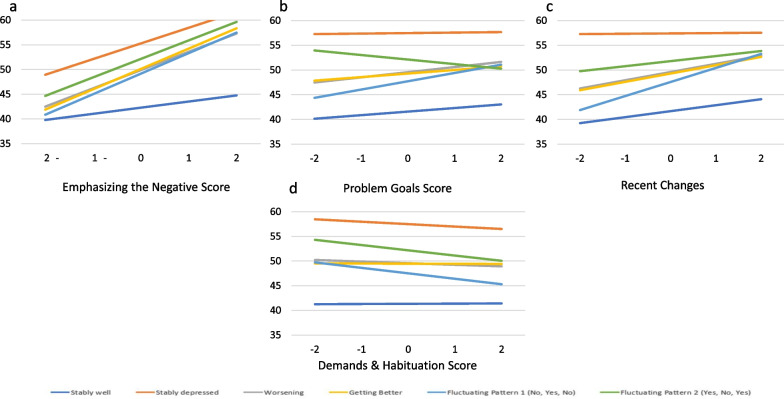
Significant Two-way Interactions for Emphasizing the Negative, Problem Goals, Recent Changes, and Demands & Habituation. These plots show adjusted means by depression-trajectory group illustrating the two-way interaction (appraisal-by-group) on Emphasizing the Negative, Problem Goals, Recent Changes, and Demands & Habituation. All trajectory groups had significant parameter estimates for Emphasizing the Negative. For all three appraisal processes, the Stably Depressed and Fluctuating Pattern 1 (No, Yes, No) had such

**Fig. 5:**
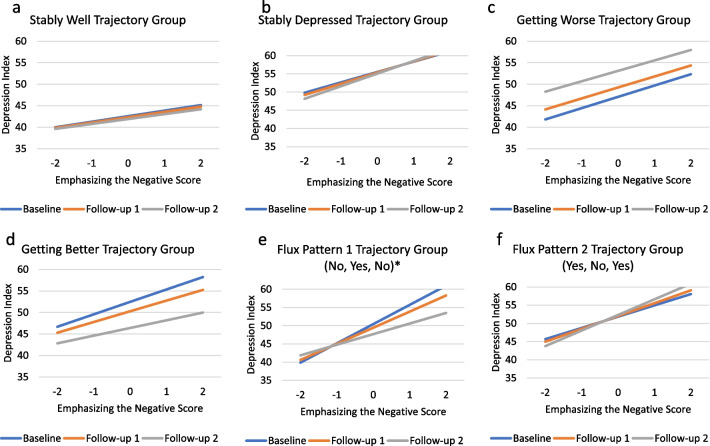
**a**–**f** Emphasizing the Negative Three-Way Interactions. These plots show adjusted means by depression-trajectory group illustrating the three-way interaction (appraisal-by-group-by-time) on Emphasizing the Negative for each data collection timepoint. Asterisks (*) indicate significant trajectory group parameter estimates

Appraisal processes characterized by emphasizing the negative had a significant main effect for appraisal, significant appraisal-by-group interactions for all groups, and a significant appraisal-by-group-by-time interaction only for the Fluctuating Pattern 1 (No, Yes, No) group. For all trajectory groups, focusing on the negative was associated with worse depression relative to the Stably Well group (Fig. 4a), and the magnitude of the association relative to the Stably Well group varied across trajectory groups and over time for the Fluctuating Pattern 1 group (Fig. 5e; Additional file 1: Table S5, Model 3b). For the Fluctuating Pattern 1 group, the association between focusing on the negative and depression weakens over time, while it remains roughly constant for the Stably Well group.

Appraisal processes characterized by focusing on problem goals had a significant main effect for appraisal and appraisal-by-group interaction, but a non-significant appraisal-by-group-by-time interaction. Specifically, among those who were Stably Well, focusing on problem goals was associated with more depression. Yet, for those who were Stably Depressed, focusing more or less on problem goals does not appear to be associated with depression compared to the Stably Well group. Similar to those who were Stably Well, focusing more on problem goals was associated with more depression for those in Fluctuating Pattern 1 (No, Yes, No), although the association appears to be stronger for the latter group (Fig. 4b; Additional file 1: Table S5, Model 4c).

**Fig. 6:**
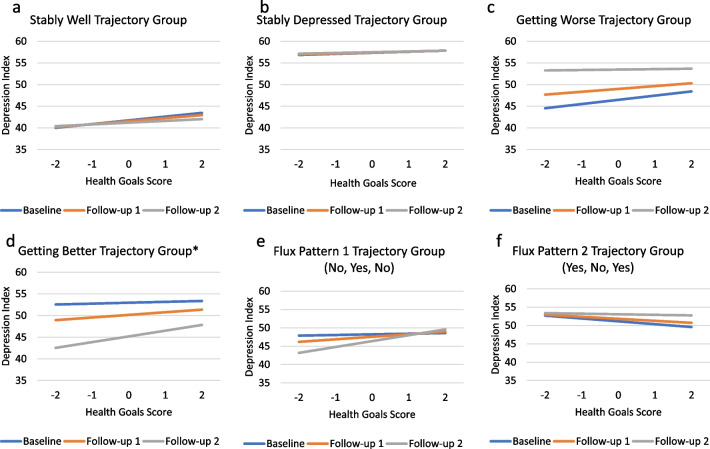
**a**–**f**. Problem Goals Three-Way Interactions. These plots show adjusted means by depression-trajectory group illustrating the three-way interaction (appraisal-by-group-by-time) on Problem Goals for each data collection timepoint. Asterisks (*) indicate significant trajectory group parameter estimates

Appraisal processes characterized by focusing on health goals had a significant main effect for appraisal, a non-significant appraisal-by-group interaction, and a significant appraisal-by-group-by-time interaction (Fig. 6a–f, Additional file 1: Table S5, Models 2d and 4d). For members of the Stably Well group, as focusing on health goals increases, the predicted depression scores also increase, but remain low relative to the other trajectory groups. Over time, the depression levels for members of the Getter Better group decreased, on average, regardless of how much they were focusing on their health goals (Fig. 6d). However, at baseline the relationship between health goals and depression was very weak but is much stronger by the second follow-up.

**Fig. 7:**
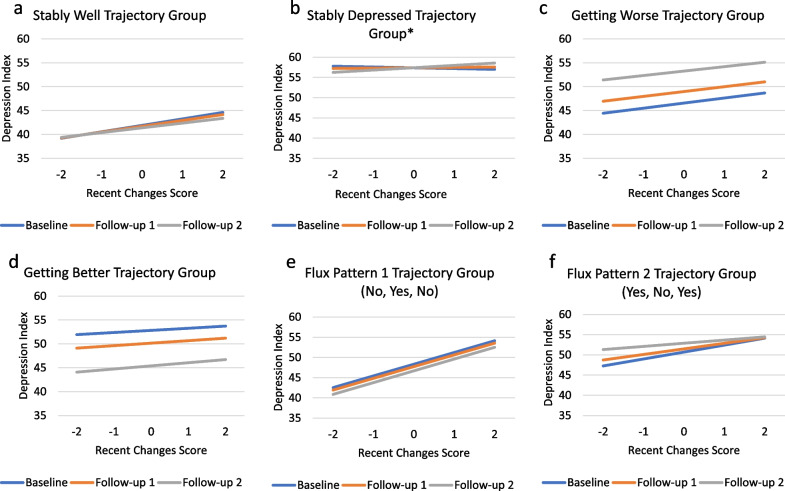
**a**–**f**. Recent Changes Three-Way Interactions. These plots show adjusted means by depression-trajectory group illustrating the three-way interaction (appraisal-by-group-by-time) on Recent Changes for each data collection timepoint. Asterisks (*) indicate significant trajectory group parameter estimates

Appraisal processes characterized by focusing on recent changes had a significant main effect for appraisal, appraisal-by-group interactions (Fig. 4c), and appraisal-by-group-by-time interactions (Fig. 7a–f). Among those who were Stably Well, increased focusing on recent changes was associated with increased depression across all three time points. For those whose depression trajectory was characterized as Stably Depressed, focusing more on recent changes was associated with more depression later in the follow-up than earlier (Fig. 7b; Additional file 1: Table S5, Model 4e). For those in the Fluctuating Pattern 1 (No, Yes, No) group, focusing on recent changes was associated with more depression relative to the Stably Well group (Fig. 4c).

Appraisal processes characterized by focusing on demands and habituation had a significant main effect for appraisal in the models that only adjusted for time and demographic covariates; and significant appraisal-by-group interactions only in the context of non-significant appraisal-by-group-by-time interactions (Additional file 1: Table S5, Model 4f). We thus examined possible suppression effects by adding group-by-time interactions to the initial two-way model to see if appraisal-by-group emerged as significant when the former was controlled. A suppression hypothesis was supported such that after adjusting for group-by-time, there was a significant appraisal-by-group interaction for the Stably Depressed and Fluctuating Pattern 1 (No, Yes, No) groups suggesting that those in these groups who focused more on demands and habituation reported less depression relative to the Stably Well group (Fig. 4d, Additional file 1: Table S5, Model 5f).

## Discussion

The present work is, to our knowledge, the first study of response-shift effects in depression. It is also the first to directly test the Appraisal Theory [23] using random-effects modeling that captures appraisal processes over time as mediated and moderated response shift. In this study during the first 15.5 months of the COVID pandemic, there were notable unadjusted trajectory-group differences in emphasizing the negative, problem goals, health goals, and recent changes. In contrast, there were less distinct trajectory-group differences in their focus on standards of comparison and demands/habituation. The multivariate models revealed that emphasizing the negative was associated with worse depression for all trajectory groups, consistent with the idea of depressive cognitions from cognitive-behavioral therapy [9]. Our results suggest that such therapy might expand its focus to cognitions related to how one thinks about QOL in addition to self-talk cognitions. For example, comparing oneself to others and emphasizing the negative were associated with worse depression across groups as compared to those who were Stably Well. Helping people to shift from such appraisal processes would be a worthwhile focus of cognitive-behavioral therapy.

There were notable differences across trajectory groups that shed further light on the complex relationship between appraisal and depression. The Stably Depressed group participants were relatively consistent in their appraisal processes over time in the unadjusted comparisons (Fig. 2). However, relative to the Stably Well group, those in the Stably Depressed group who increasingly focused on recent changes, or decreasingly on comparing themselves to others, did somewhat worse over time. Further, those in the Stably Depressed group who emphasized the negative and focused less on problem goals or on demands/habituation reported more depression relative to those who were Stably Well. This suggests that even within a “stable” group, how one thinks about one’s life can change their levels of depression in statistically significant ways. Over a more extended time, it is possible that these small changes could instigate a person to move into the Getting Better group. We also found that focusing on problem goals and on recent changes was associated with increased depression among those in the Fluctuating Pattern 1 (No, Yes, No) group. Among those who were Getting Better, at baseline, the average depression score was roughly stable regardless of how much this group focused on health goals. However, by Follow-up 2, increasingly focusing on health goals was associated with worse depression. Thus, egregious life circumstances may play less of a role in longitudinal levels of depression for the Stably Depressed, perhaps suggesting a more endogenous rather than reactive type of depression [59, 60]. In contrast, such circumstances seem to play more of a role for people who have transient periods of depression as well as for those with improving trajectories.

By examining the intersection of individual depression trajectories with appraisal processes, this study begins the investigation of response-shift effects as a function of depression. It has long been hypothesized that depression renders one unable to adapt (i.e., unable to make response shifts in the face of health-state changes) [21]. Our findings do suggest that not only was the Stably Depressed group particularly consistent in their focus on negative aspects of their life, but also that other groups tended to focus on these same negative aspects when they were depressed (e.g., the Fluctuating Pattern 1 (No, Yes, No). Further, two of the four significant three-way interactions (i.e., group-by-appraisal-by-time) concerned the Stably Depressed group. Thus, the relationship between these appraisal processes and depression changed over time for this group in particular, as compared to the Stably Well group. For example, focusing on comparing oneself to others was associated with worse depression at baseline and less depression at follow-up among the Stably Depressed. In contrast, focusing on recent changes became more detrimental over follow-up for this group in particular. Yet, in both cases, depression remained overall high, so actual fluctuations were relatively small. This demonstrates how appraisal measures can be sensitive to relatively small changes, which can be useful in clinical interventions. It is possible these findings suggest a maladaptive response-shift effect in the face of the COVID-19 pandemic. Future research might utilize qualitative research methods to better understand the nature of these changes and what they reveal about response shift among those who are Stably Depressed.

The present study has several advantages, including a robust sample that was selected to represent the general United States population on age, gender, region, and income; utilization of a depression indicator with similar content to that of a prominent depression screener; and longitudinal analysis that to some degree enables testing of causal hypotheses. Further, by dint of collecting data during the first 15.5 months of the COVID pandemic, the study highlights the context of COVID. For instance, people probably spent more time alone during COVID than in general, giving them more time to ruminate which may have exacerbated depression. They might also have had less access to mental health care. The study findings thus highlight the importance of strengthening mental health care systems in periods of global crisis.

### Limitations

The study’s limitations should, however, be acknowledged. First, while our findings implicate appraisal processes in depression trajectories, there may well be other variables not included that are relevant to the research question, such as whether the person is participating in a treatment for depression and what type of treatment [61]; exercise [62] and lifestyle [63] factors, including reserve-building activities [12]; social capital at the structural (network structure, civic engagement, trust), relational (social networks, social cohesion), and cognitive (norms and values) levels [64]; and natural sleep habits related to circadian rhythm [65]. Thus, despite the content-rich longitudinal data, one cannot make unequivocal causal statements, but rather can generate hypotheses in the context of a quasi-experimental study design. Second, the generalizability of the study’s findings to non-pandemic times would need to be examined in independent research using similar measures and longitudinal design. Third, the operationalization of depression relied on using items similar to the PHQ-8 from various instruments to construct a new score that is ‘fit-for-purpose’. Future research might replicate the present study using the PHQ-8 or an external clinician to measure or assess depression. Fourth, although the data analysis utilized all available data and was empowered by having multiple data points for each person, it is possible that non-significant three-way interactions could be attributed to being underpowered to detect such. This may be especially true for the smaller trajectory groups. The three-way interactions that we did detect would therefore have been particularly large effects. It is difficult to get a sense of the effect size with such small unstandardized coefficients, as the coefficient units for time are in days. Fifth, with only three time points modeled and not having a more formal assessment of depression at each time point (i.e., PHQ-8), the analyses may be limited by measurement error or noise. Future work might extend the present work by collecting cognitive-appraisal data at many more time points, and characterizing depression more formally using the PHQ-8. Finally, the present work considers the COVID pandemic as a background catalyst. Subsequent work building on this study has addressed how cognitive-appraisal processes buffered the impact of COVID-specific stressors and resources on depressive symptoms [66].

## Conclusions

In summary, this study is the first to investigate appraisal processes implicated in depression trajectories over time. Our results suggest that during these first 15.5 months of the COVID pandemic, Stably Depressed people consistently focused on negative aspects of their life, and that the relationship between these appraisal processes and depression changed over time for this group in particular, implicating response-shift phenomena. Further, other groups engaged in similar negative appraisal processes when they were depressed. Thus, our findings lend further support to the idea that how one thinks about QOL has an impact on one’s mental health. Cognitive-behavioral interventions might expand the target of the self-talk to embrace such health-specific appraisal processes.

## Supplementary Information

**Additional file 1**. Supplementary Tables 1–5.

**Additional file 2**. **Figure S1**: Receiver Operating Characteristic Curve for the Depression Index. This analysis supported the use of a single factor score as a depression index (Area Under the Curve = 0.78, 95% confidence interval: 0.75–0.81). A score of 50 on our Depression index was associated with a high sensitivity or true-positive rate of 0.71, and relatively low false-positive rate (i.e., 1 minus specificity) of 0.30. The dashed black intersecting lines indicate this (0.3, 0.7) coordinate associated with this cut-point. **Figure S2**: **a**–**f**. Depression Trajectory Groups. These plots illustrate the individual growth trajectories for the six depression trajectory groups by showing a random selection of 20 patients within each group.

## Data Availability

The study data are confidential and thus not able to be shared.
