# Supplementary material for: Depression trajectories during the COVID-19 pandemic: a secondary analysis of the impact of cognitive-appraisal processes
Source: J Patient Rep Outcomes. 2023 Jul 13;7:67. doi: 10.1186/s41687-023-00600-z (PMC10344849; doi:10.1186/s41687-023-00600-z)
Supplement: Supplementary file 2 — Additional file 2. Figure S1: Receiver Operating Characteristic Curve for the Depression Index. This analysis supported the use of a single factor score as a depression index (Area Under the Curve = 0.78, 95% confidence interval: 0.75–0.81). A score of 50 on our Depression index was associated with a high sensitivity or true-positive rate of 0.71, and relatively low false-positive rate (i.e., 1 minus specificity) of 0.30. The dashed black intersecting lines indicate this (0.3, 0.7) coordinate associated with this cut-point. Figure S2: a–f. Depression Trajectory Groups. These plots illustrate the individual growth trajectories for the six depression trajectory groups by showing a random selection of 20 patients within each group. [file 41687_2023_600_MOESM2_ESM.pptx]

## Slide 1
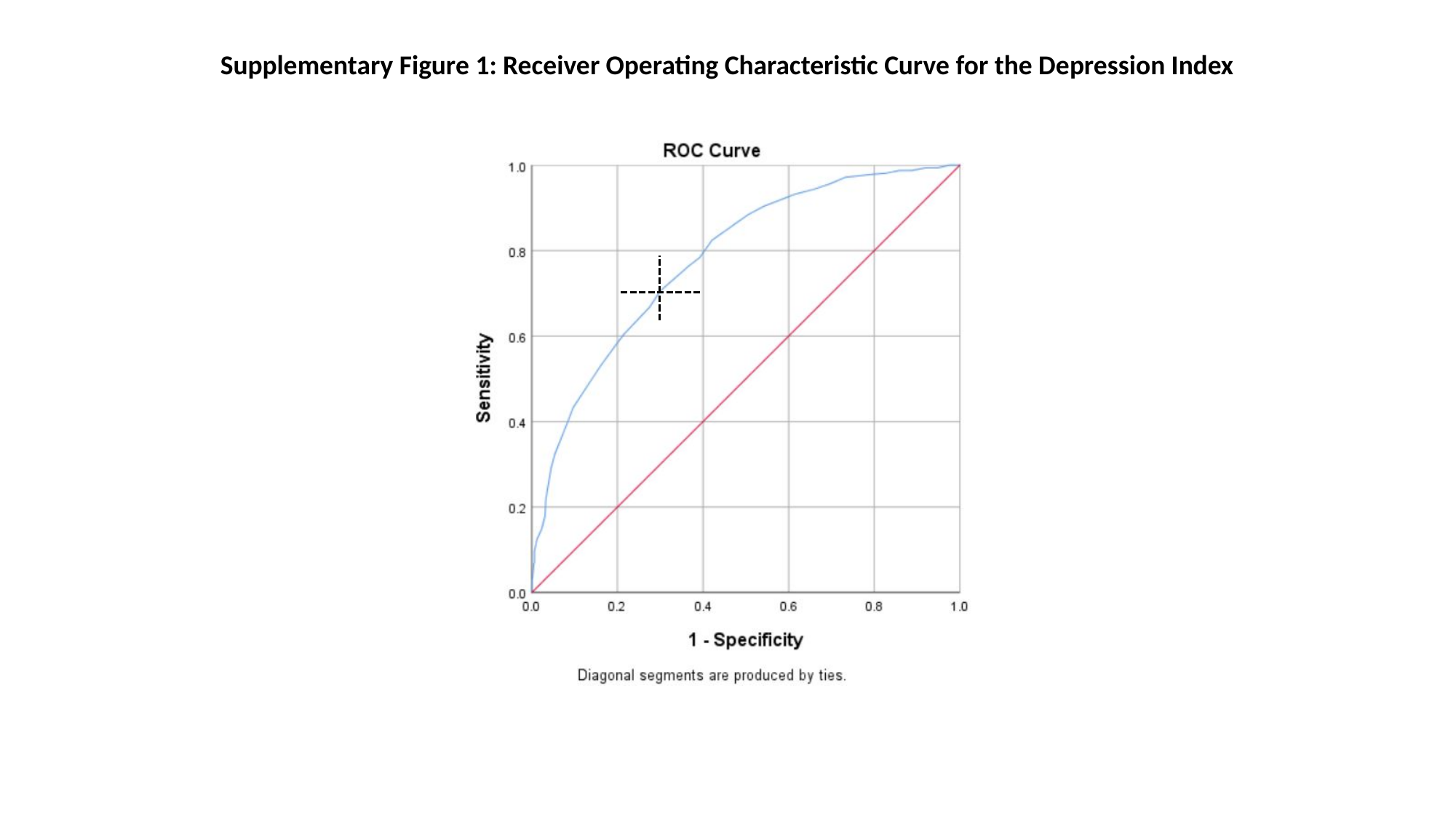

Supplementary Figure 1: Receiver Operating Characteristic Curve for the Depression Index

## Slide 2
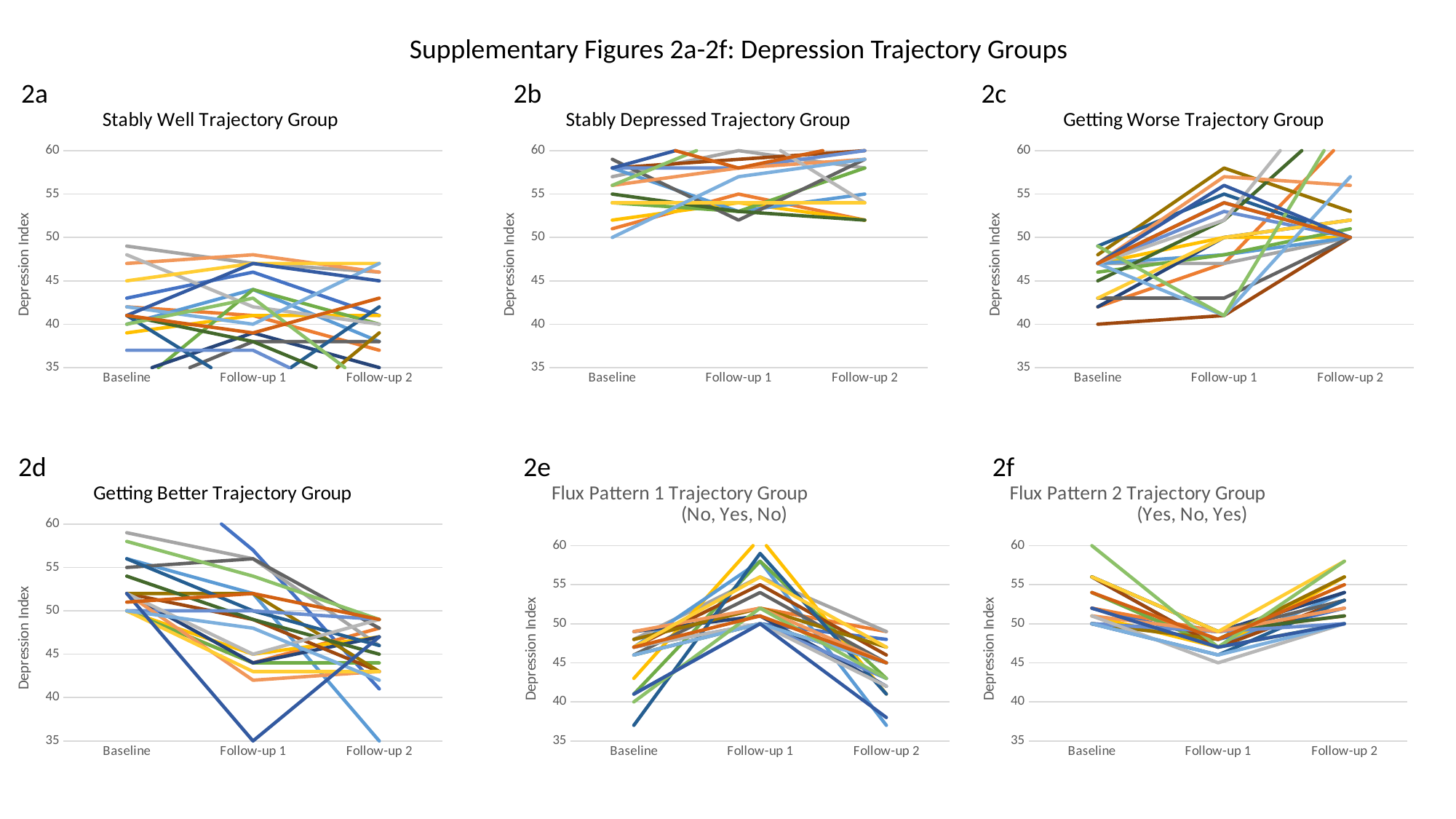

Supplementary Figures 2a-2f: Depression Trajectory Groups
2a
2b
2c
### Chart: Stably Well Trajectory Group
| Category | | | | | | | | | | | | | | | | | | | | |
|---|---|---|---|---|---|---|---|---|---|---|---|---|---|---|---|---|---|---|---|---|
| Baseline | 43.0 | 42.0 | 49.0 | 39.0 | 40.0 | 32.0 | 34.0 | 35.0 | 32.0 | 35.0 | 41.0 | 41.0 | 37.0 | 47.0 | 48.0 | 45.0 | 42.0 | 40.0 | 41.0 | 41.0 |
| Follow-up 1 | 46.0 | 41.0 | 47.0 | 41.0 | 44.0 | 44.0 | 39.0 | 27.0 | 38.0 | 27.0 | 32.0 | 38.0 | 37.0 | 48.0 | 42.0 | 47.0 | 40.0 | 43.0 | 47.0 | 39.0 |
| Follow-up 2 | 41.0 | 37.0 | 46.0 | 41.0 | 38.0 | 40.0 | 35.0 | 27.0 | 38.0 | 39.0 | 42.0 | 32.0 | 30.0 | 46.0 | 40.0 | 47.0 | 47.0 | 32.0 | 45.0 | 43.0 |
### Chart: Stably Depressed Trajectory Group
| Category | | | | | | | | | | | | | | | | | | | | |
|---|---|---|---|---|---|---|---|---|---|---|---|---|---|---|---|---|---|---|---|---|
| Baseline | 71.0 | 51.0 | 57.0 | 52.0 | 58.0 | 54.0 | 62.0 | 58.0 | 59.0 | 68.0 | 60.0 | 55.0 | 58.0 | 56.0 | 61.0 | 54.0 | 50.0 | 56.0 | 58.0 | 62.0 |
| Follow-up 1 | 72.0 | 55.0 | 60.0 | 54.0 | 53.0 | 53.0 | 61.0 | 59.0 | 52.0 | 71.0 | 66.0 | 53.0 | 58.0 | 58.0 | 63.0 | 54.0 | 57.0 | 62.0 | 62.0 | 58.0 |
| Follow-up 2 | 68.0 | 52.0 | 58.0 | 52.0 | 55.0 | 58.0 | 60.0 | 60.0 | 59.0 | 73.0 | 65.0 | 52.0 | 60.0 | 59.0 | 54.0 | 54.0 | 59.0 | 63.0 | 62.0 | 61.0 |
### Chart: Getting Worse Trajectory Group
| Category | | | | | | | | | | | | | | | | | | | | |
|---|---|---|---|---|---|---|---|---|---|---|---|---|---|---|---|---|---|---|---|---|
| Baseline | 47.0 | 42.0 | 47.0 | 47.0 | 47.0 | 46.0 | 42.0 | 40.0 | 43.0 | 48.0 | 49.0 | 45.0 | 47.0 | 47.0 | 47.0 | 43.0 | 47.0 | 49.0 | 47.0 | 47.0 |
| Follow-up 1 | 54.0 | 47.0 | 47.0 | 50.0 | 48.0 | 48.0 | 50.0 | 41.0 | 43.0 | 58.0 | 55.0 | 52.0 | 53.0 | 57.0 | 52.0 | 50.0 | 41.0 | 41.0 | 56.0 | 54.0 |
| Follow-up 2 | 50.0 | 62.0 | 50.0 | 50.0 | 50.0 | 51.0 | 52.0 | 50.0 | 50.0 | 53.0 | 50.0 | 65.0 | 50.0 | 56.0 | 70.0 | 52.0 | 57.0 | 65.0 | 50.0 | 50.0 |2d
2e
2f
### Chart: Getting Better Trajectory Group
| Category | | | | | | | | | | | | | | | | | | | | |
|---|---|---|---|---|---|---|---|---|---|---|---|---|---|---|---|---|---|---|---|---|
| Baseline | 69.0 | 50.0 | 59.0 | 50.0 | 56.0 | 50.0 | 52.0 | 52.0 | 55.0 | 52.0 | 56.0 | 54.0 | 50.0 | 52.0 | 52.0 | 50.0 | 50.0 | 58.0 | 52.0 | 51.0 |
| Follow-up 1 | 57.0 | 44.0 | 56.0 | 45.0 | 52.0 | 44.0 | 44.0 | 49.0 | 56.0 | 52.0 | 50.0 | 49.0 | 50.0 | 42.0 | 45.0 | 43.0 | 48.0 | 54.0 | 35.0 | 52.0 |
| Follow-up 2 | 41.0 | 48.0 | 46.0 | 47.0 | 35.0 | 44.0 | 47.0 | 43.0 | 48.0 | 43.0 | 46.0 | 45.0 | 49.0 | 43.0 | 49.0 | 43.0 | 42.0 | 49.0 | 47.0 | 49.0 |
### Chart: Flux Pattern 1 Trajectory Group (No, Yes, No)
| Category | | | | | | | | | | | | | | | | | | | | |
|---|---|---|---|---|---|---|---|---|---|---|---|---|---|---|---|---|---|---|---|---|
| Baseline | 47.0 | 48.0 | 48.0 | 43.0 | 47.0 | 41.0 | 49.0 | 47.0 | 46.0 | 48.0 | 37.0 | 46.0 | 41.0 | 49.0 | 47.0 | 47.0 | 46.0 | 40.0 | 41.0 | 47.0 |
| Follow-up 1 | 50.0 | 52.0 | 56.0 | 61.0 | 58.0 | 58.0 | 51.0 | 55.0 | 54.0 | 52.0 | 59.0 | 50.0 | 50.0 | 52.0 | 50.0 | 56.0 | 50.0 | 52.0 | 50.0 | 51.0 |
| Follow-up 2 | 48.0 | 49.0 | 49.0 | 41.0 | 37.0 | 43.0 | 42.0 | 46.0 | 45.0 | 47.0 | 41.0 | 45.0 | 43.0 | 45.0 | 42.0 | 47.0 | 45.0 | 43.0 | 38.0 | 45.0 |
### Chart: Flux Pattern 2 Trajectory Group (Yes, No, Yes)
| Category | | | | | | | | | | | | | | | | | | | | |
|---|---|---|---|---|---|---|---|---|---|---|---|---|---|---|---|---|---|---|---|---|
| Baseline | 52.0 | 52.0 | 50.0 | 51.0 | 56.0 | 54.0 | 50.0 | 56.0 | 56.0 | 50.0 | 50.0 | 56.0 | 50.0 | 51.0 | 51.0 | 56.0 | 50.0 | 60.0 | 52.0 | 54.0 |
| Follow-up 1 | 48.0 | 49.0 | 48.0 | 47.0 | 47.0 | 47.0 | 49.0 | 47.0 | 49.0 | 48.0 | 46.0 | 49.0 | 49.0 | 49.0 | 45.0 | 49.0 | 46.0 | 47.0 | 47.0 | 48.0 |
| Follow-up 2 | 52.0 | 51.0 | 56.0 | 56.0 | 54.0 | 50.0 | 54.0 | 53.0 | 53.0 | 56.0 | 53.0 | 51.0 | 50.0 | 52.0 | 50.0 | 58.0 | 50.0 | 58.0 | 50.0 | 55.0 |
